# Lung Cancer Incidence Trends Among Men and Women — United States, 2005–2009

**Published:** 2014-01-10

**Authors:** S. Jane Henley, Thomas B. Richards, J. Michael Underwood, Christie R. Eheman, Marcus Plescia, Timothy A. McAfee

**Affiliations:** 1 Div of Cancer Prevention and Control; 2 Office on Smoking and Health, National Center for Chronic Disease Prevention and Health Promotion, CDC

Lung cancer is the leading cause of cancer death and the second most commonly diagnosed cancer (excluding skin cancer) among men and women in the United States (1,2). Although lung cancer can be caused by environmental exposures, most efforts to prevent lung cancer emphasize tobacco control because 80%–90% of lung cancers are attributed to cigarette smoking and secondhand smoke (1). One sentinel health consequence of tobacco use is lung cancer (1,2), and one way to measure the impact of tobacco control is by examining trends in lung cancer incidence rates, particularly among younger adults (3). Changes in lung cancer rates among younger adults likely reflect recent changes in risk exposure (3). To assess lung cancer incidence and trends among men and women by age group, CDC used data from the National Program of Cancer Registries (NPCR) and the National Cancer Institute’s Surveillance, Epidemiology, and End Results (SEER) program for the period 2005–2009, the most recent data available. During the study period, lung cancer incidence decreased among men in all age groups except <35 years and decreased among women aged 35–44 years and 54–64 years. Lung cancer incidence decreased more rapidly among men than among women and more rapidly among adults aged 35–44 years than among other age groups. To further reduce lung cancer incidence in the United States, proven population-based tobacco prevention and control strategies should receive sustained attention and support (4).

Data on new cases of invasive lung cancer (*International Classification of Diseases for Oncology, Third Edition*: C34.0–C34.9) diagnosed during the most recent 5-year period with available data (2005–2009) were obtained from population-based cancer registries affiliated with the NPCR and SEER programs, which when combined cover the entire U.S. population. Data from all cancer registries that met the United States Cancer Statistics (USCS) data-quality criteria for each year during 2005–2009 were used in this report.*

Population denominators for incidence rates were race/ethnicity-specific and sex-specific county population estimates from the 2000 U.S. Census, as modified by SEER and aggregated to state and national levels.† Annual incidence rates per 100,000 population were age-adjusted (using 19 age groups) by the direct method to the 2000 U.S. standard population.§ Annual percentage change (APC) was used to quantify the change in incidence rates over time and was calculated using least-squares regression. Rates were considered to increase or decrease if p<0.05; otherwise rates were considered stable. The rate ratio (RR) of incidence among women to men was calculated (Table 1). Lung cancer incidence rates and trends were analyzed for men and women separately by age group for the United States and by U.S. Census region and state.

During 2005–2009, a total of 569,366 invasive lung cancer cases among men and 485,027 among women were reported in the United States (Table 1). Lung cancer incidence was highest among those aged ≥75 years and decreased with decreasing age (Figure). In all age groups except persons aged <35 years and 35–44 years, lung cancer incidence rates were higher among men than among women; this difference was greatest among those aged ≥75 years and narrowed with decreasing age (Table 1). From 2005 to 2009, lung cancer incidence decreased among men in all age groups except those aged <35 years, with an APC of −2.6% overall; among women, lung cancer incidence decreased among those aged 35–44 and 55–64 years and was stable in all other age groups yielding an APC of −1.1% overall (Table 1). Lung cancer incidence rates decreased most rapidly among adults aged 35–44 years, decreasing 6.4% per year among men and 5.9% per year among women (Table 1).

Lung cancer incidence decreased to a statistically significant extent from 2005 to 2009 among men in all U.S. Census regions and 23 states, and among women in the South and West U.S. Census regions and seven states (Table 2). By state and age group, lung cancer incidence rates decreased or were stable in most states (Table 2).

## Editorial Note

CDC has declared reducing tobacco use a “winnable battle”¶ and supports comprehensive efforts to prevent the initiation of tobacco use, promote quitting, and ensure smoke–free environments. This report documents recent decreases in lung cancer incidence during 2005–2009 in the United States, with lung cancer incidence declining more rapidly among men compared with women in all age groups except age <35 years. Since 1964 when the first Surgeon General’s report on the health consequences of smoking was published, cigarette smoking cessation rates increased and cigarette smoking initiation rates decreased more rapidly among men than women (5). As a result, cigarette smoking behaviors have become more similar among men and women, especially among those in recent birth cohorts (5). Subsequently, the gap in lung cancer between men and women has been reported to be diminishing (6). This report shows that differences in lung cancer incidence between men and women narrowed with decreasing age, and that among adults aged <45 years, men had slightly lower rates of lung cancer than women.

What is already known on this topic?

Because of shifts in cigarette smoking prevalence, the gap between men and women in lung cancer incidence is diminishing, particularly among younger adults.

What is added by this report?

From 2005 to 2009, lung cancer incidence rates decreased among men and women in the United States overall, among men in 23 states, and among women in seven states. Lung cancer incidence rates during 2005–2009 decreased more rapidly among men than among women and more rapidly among adults aged 35–44 years than among other age groups. As a result, differences in lung cancer incidence between men and women narrowed with decreasing age; among adults aged 35–44 years, men had slightly lower rates of lung cancer than did women.

What are the implications for public health practice?

Although lung cancer incidence is decreasing overall, it is not decreasing at the same pace among men and women, nor in all age groups, and it is not decreasing in all states. Continued attention to local, state, and national population-based tobacco prevention and control strategies are needed to achieve further reductions in tobacco use among men and women of all ages to reduce lung cancer in the United States.

Another finding is that lung cancer incidence decreased most rapidly from 2005 to 2009 among men and women aged 35–44 years compared with other age groups. Although many factors might have contributed to this decline, a study of 44 states showed that strong tobacco control indicators were correlated with lower lung cancer incidence rates among adults age 20–44 years (7). Whereas a coordinated, multicomponent approach to tobacco prevention and control is needed to reduce tobacco use, younger adults might be more sensitive than older adults to certain interventions like increased tobacco prices. A systematic review of tobacco control interventions found that for every 10% increase in cigarette prices, cigarette smoking prevalence decreased 1%–14% among youth compared with 1%–4.5% among adults (8). In 2010, a higher proportion of adults aged 18–24 years attempted to quit smoking cigarette and succeeded in quitting than adults aged 45–64 years (9). Other population-based strategies proven to reduce tobacco use among youths and adults include comprehensive smoke-free laws, restriction of tobacco advertising and promotion, and mass media campaigns (4,8). These strategies are effective in changing the cigarette smoking behavior of both men and women, and can be combined with individual-based strategies such as providing access to telephone quitlines and health-care coverage for tobacco cessation treatments (4,8). Additionally, strategies for tobacco control have expanded now that the Food and Drug Administration has been granted the authority to regulate the manufacture, distribution, and marketing of tobacco products.**

In 2007, CDC updated recommendations regarding the estimated minimum level of funding needed to implement and sustain statewide comprehensive tobacco control programs; the combined recommended amount across programs was $3.7 billion.†† In contrast, in 2010, states appropriated only $0.64 billion, amounting to 2.4% of their state tobacco revenues, for tobacco control (10). A previous report showed that states varied substantially in their success at reducing cigarette smoking prevalence and lung cancer incidence (2). This report shows that lung cancer incidence decreased during 2005–2009 among men in all U.S. Census regions and 23 states, and decreased among women in the South and West and seven states; lung cancer incidence rates were stable in all other states. Another finding is that lung cancer incidence rates during 2005–2009 stabilized among women aged 45–54 years. This age group includes women born during 1950–1960 who were young adults during an era in which cigarettes were aggressively marketed toward women (1). This generation experienced a high prevalence of cigarette smoking as young women and high rates of lung cancer mortality as older women (1).

The findings in this report are subject to at least four limitations. First, populations were estimated from the 2000 Census by the U.S. Census Bureau; errors in these estimates might increase as time passes after the census, leading to underestimates or overestimates of incidence rates. Second, analyses based on race and ethnicity might be biased if race and ethnicity were misclassified; efforts were made to ensure that this information was as accurate as possible.§§ Third, delays in cancer reporting might result in an underestimate of the incidence rate. Fourth, analyses of trends should be carefully interpreted; some rates might be actually increasing or decreasing although the trend is not statistically significant..

From 2005 to 2009 lung cancer incidence rates decreased among men and women in the United States overall, more rapidly among men than among women, and more rapidly among adults aged 35–44 years than among other age groups. As a result, differences in lung cancer incidence between men and women narrowed with decreasing age. However, continued attention and support to proven population-based tobacco prevention and control strategies will be needed to reduce tobacco use among both men and women and further reduce lung cancer in the United States (4).

## Figures and Tables

**FIGURE:**
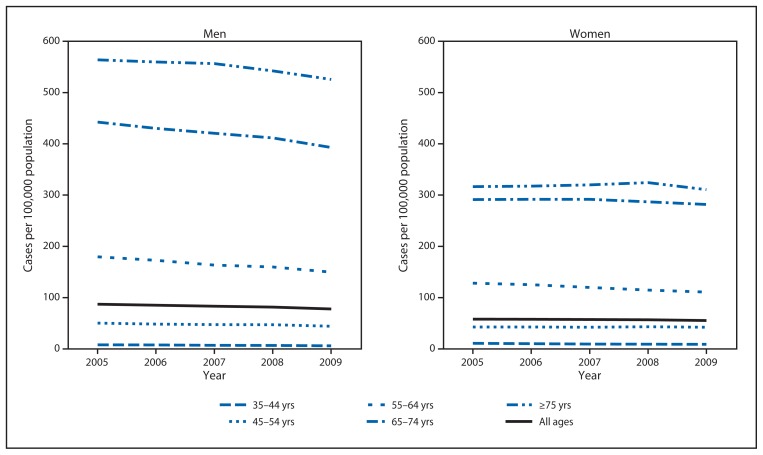
Rate* of invasive lung cancer cases among men and women, by age group — United States, 2005–2009 **Sources:** CDC’s National Program of Cancer Registries and National Cancer Institute’s Surveillance, Epidemiology, and End Results program. * Lung cancer incidence per 100,000.

**TABLE 1:** Number of invasive lung cancer cases, average annual rate,* and annual percentage change (APC), by sex and age group — United States, 2005–2009

Age group (yrs)	Men			Women			
	
	No.	Rate	APC (%)	No.	Rate	RR women to men	APC (%)
**Overall**	**569,366**	**82.9**	**−2.6** †	**485,027**	**55.7**	**0.7**	**−1.1** †
<35	1,239	0.3	0.4	1,299	0.4	1.3	1.0
35–44	7,675	7.1	−6.4†	8,539	7.9	1.1	−5.9†
45–54	51,409	47.5	−2.9†	45,999	41.2	0.9	−0.1
55–64	128,474	164.4	−4.3†	100,024	118.7	0.7	−3.8†
65–74	186,161	418.8	−2.8†	152,064	289.5	0.7	−0.8
≥75	194,408	549.4	−1.7†	177,102	319.1	0.6	−0.2

**Abbreviation:** RR = rate ratio.

**Sources:** CDC’s National Program of Cancer Registries and National Cancer Institute’s Surveillance, Epidemiology, and End Results program.

* Per 100,000 persons, age-adjusted to the 2000 U.S. standard population.

† APC trend was significant at p<0.05.

**TABLE 2:** Annual percentage change (APC) in invasive lung cancer incidence, by sex, age group (yrs), U.S. Census region, and state — United States, 2005–2009

Region/State	Men						Women					
	
	All ages APC (%)	35–44 APC (%)	45–54 APC (%)	55–64 APC (%)	65–74 APC (%)	≥75 APC (%)	All ages APC (%)	35–44 APC (%)	45–54 APC (%)	55–64 APC (%)	65–74 APC (%)	≥75 APC (%)
**United States**	**−2.6** *	**−6.5** *	**−2.9** *	**−4.3** *	**−2.7** *	**−1.7** *	**−1.1** *	**−5.8** *	**−0.1**	**−3.7** *	**−0.8**	**−0.1**
**Northeast**	−2.1*	−3.7	−3.1*	−4.6*	−2.2*	−0.9	−0.5	−6.1*	−0.1	−2.9*	−0.3	0.9
Connecticut	−1.5	—†	−3.5	−2.8	−3.0	0.3	−0.9	—	−1.9	−4.0	−1.2	1.4
Maine	−2.3	—	0.1	−4.6	−1.3	−3.2	0.4	—	6.9	−3.6	0.8	0.2
Massachusetts	−3.6*	−6.9	0.4	−6.1*	−3.8	−3.0*	−1.4	−0.3	1.2	−4.5	−0.8	−1.1
New Hampshire	−2.8	—	2.7	−7.2*	−2.6	−2.2	−2.8	—	−1.6	−6.6*	−4.2	0.9
New Jersey	−3.3*	−4.6	−7.1*	−4.5*	−3.5	−2.1*	−1.3	−11.1*	−2.0	−3.5*	−0.5	0.0
New York	−1.0	−5.9*	−3.5*	−3.7*	−1.1	0.9	0.0	−6.3*	−0.4	−3.2*	−0.1	2.5
Pennsylvania	−2.3*	−0.8	−3.4*	−5.2*	−1.7	−1.4	−0.1	−6.0	0.7	−1.1	0.2	0.5
Rhode Island	−1.6	—	3.8	−4.3	−2.9	0.3	1.0	—	−1.1	−1.1	1.8	2.6
Vermont	−3.0	—	−3.4	−7.7	−5.0	0.4	−1.0	—	3.7	1.4	−2.3	0.0
**Midwest**	−2.9*	−4.2*	−2.0	−4.2*	−3.0*	−2.3*	−0.8	−4.5*	1.8	−3.9*	−0.6	0.2
Illinois	−1.9*	−6.1	−3.3	−3.8*	−2.1	−0.6	0.6	−6.8*	2.1*	−3.2*	1.5*	1.5
Indiana	−2.3	−3.0	−1.6	−1.3	−3.3	−2.1	−0.9	−2.0	1.2	−2.7	−0.4	−1.0
Iowa	−2.2	—	1.1	−4.8	−0.8	−3.1*	0.1	—	4.4	−2.5	0.5	−0.2
Kansas	−3.1	—	1.8	−2.7	−1.3	−5.8	−0.5	—	3.6	−6.5*	−1.5	3.7
Michigan	−3.2*	−5.8	−2.0	−2.9*	−4.2*	−2.7	−2.4	−11.1*	−2.1	−4.4	−2.1	−1.3
Minnesota	−1.6	−7.7	0.5	−5.5	−0.4	−1.2	1.0	−2.8	2.9	−3.3	1.7	2.6
Missouri	−3.5*	−4.4	−2.5	−5.8*	−4.8*	−1.5	−1.1	1.2	4.8	−2.8	−2.5	−0.8
Nebraska	−4.6*	—	3.3	−7.3	−6.6	−2.3	−0.8	—	15.6	−8.5	−0.2	−0.9
North Dakota	−4.6*	—	—	−9.9	−5.9	−3.3	−1.7	—	—	−3.4	−1.7	4.8
Ohio	−3.4*	−2.3	−3.7	−5.0*	−3.0	−3.2*	−1.2*	−3.1	1.9*	−4.0	−1.2*	−0.5
South Dakota	−5.0	—	−0.7	−5.9*	0.0	−8.3	−2.5*	—	—	−16.6*	−2.0	3.8
**South**	−2.8*	−9.4*	−2.8*	−4.1*	−2.5*	−2.1*	−1.3*	−6.7*	−0.2	−3.7*	−0.6	−0.6
Alabama	−0.3	−13.5*	−0.1	0.3	−1.9	1.5	0.3	−3.6	−1.4	−0.6	0.5	1.6
Arkansas	−2.9	—	−0.1	−5.2*	−1.0	−3.3	−1.5	—	0.0	−6.5*	0.3	0.2
Delaware	−3.6*	—	−3.4	−7.4*	−3.7	−2.5	−5.9*	—	0.6	−15.5*	−2.7	−4.3
District of Columbia	−2.1*	—	−0.1	−1.6	−1.4	−3.0	−2.4	—	—	−3.4	−0.9	4.4
Florida	−3.3*	−8.2*	−4.9*	−5.1*	−2.6*	−2.4*	−1.5*	−5.4	−3.1	−3.7*	−0.1	−0.8
Georgia	−2.0*	−4.6	−2.4	−3.6*	−2.2	−0.9	0.0	−7.8	0.1	−1.5	1.6	−0.3
Kentucky	−1.6	−12.9	2.2	−2.8*	−1.5	−1.3	−0.1	0.2	2.8*	−1.0	−0.9	0.5
Louisiana	−2.8	−2.3	−1.6	−3.5	−1.6	−3.7	−1.0	−8.4	−0.4	−2.2	−1.6	0.7
Maryland	−4.6	−14.9	−3.1	−3.7	−7.4*	−2.7	−2.2	−17.5*	−2.5	−5.6*	−1.7	−0.1
Mississippi	0.9	−8.7	−2.1	−0.7	−0.5	4.5	1.0	—	1.0	−5.4	3.4	3.8
North Carolina	−2.7*	−11.3*	−3.2	−3.0*	−1.1	−3.4*	−0.9	−2.1	0.4	−4.3*	0.6	−0.8
Oklahoma	−2.4	—	0.4	−4.7	−0.8	−2.3	−2.0	—	−0.4	−8.3*	0.5	−1.2*
South Carolina	−3.8*	−7.1	−4.0*	−4.8*	−3.3	−3.6	−2.2	−13.6	3.8*	−4.7*	−2.1	−1.9
Tennessee	−2.9*	−2.3	−1.3	−3.6*	−3.8*	−2.2	0.1	−6.5	1.5	−2.0	0.0	1.9
Texas	−3.5*	−10.1*	−5.3	−6.0*	−2.4	−2.9*	−2.4*	−7.8	0.2	−3.7*	−2.9*	−1.6
Virginia	−2.6*	−8.1	−2.4*	−3.8*	−3.8*	−0.9	−1.1	−4.2	0.6	−4.3	−0.5	−0.4
West Virginia	−3.6*	—	−2.1	−4.5	−3.6	−3.7*	−2.6	—	5.1*	−2.2	−3.1	−5.4
**West**	−2.6*	−4.4*	−4.4*	−4.5*	−3.5*	−0.9	−1.8*	−4.2	−2.5*	−4.0*	−1.7	−0.7
Alaska	−0.5	—	−0.2	−3.7	−6.4	6.4*	−7.2	—	1.6	−3.6	−6.7	−12.0
Arizona	−1.7	—	−7.6*	−4.4	−1.8	0.9	−1.4	4.0	−3.6	−5.4*	−0.6	0.1
California	−2.7*	−5.7	−5.2*	−4.6*	−4.1*	−0.8	−1.8	−1.9	−5.2*	−4.2*	−1.7	−0.4
Colorado	−0.1	—	−2.2	−1.0	−0.6	0.9	−0.1	—	4.3	−2.7	0.5	0.3
Hawaii	−1.9	—	−6.8	−6.0	−2.2	1.3	−1.8	—	−7.9	3.2	−5.7	1.1
Idaho	−3.8	—	6.9	−3.7	−4.3	−4.3	−3.6	—	−3.3	−7.7	−2.7	−1.2
Montana	−3.8*	—	−3.3	0.4	−7.6*	−3.0	−3.8*	—	−5.7	−8.3	−0.7	−4.8
Nevada	−3.5	—	−6.4	−5.2*	−4.1	−2.2	−1.7	—	−1.0	−1.6	1.7	−4.2*
New Mexico	−4.2*	—	−4.3	−9.9	−3.2	−3.0	−0.2	—	12.3*	−2.9	−2.5	−0.2
Oregon	−4.0*	—	−5.9	−4.1	−3.2	−4.3*	−2.0*	—	2.2	−4.4*	−3.7	−0.1
Utah	−2.2	—	−1.6	−9.6	1.7	−2.5	−4.4	—	—	1.0	−8.5	−4.7
Washington	−2.3	−3.8	−0.6	−4.0	−4.8	0.0	−2.1	−5.1	−1.2	−4.0*	−2.4	−1.1
Wyoming	2.3	—	—	−4.8	1.8	6.5	−3.6	—	—	−13.8	−4.6	1.7

**Sources:** CDC’s National Program of Cancer Registries and National Cancer Institute’s Surveillance, Epidemiology, and End Results program.

* APC trend was significant at p<0.05.

† APC is not presented for groups with <16 cases in any year. Data for Wisconsin were suppressed at the state’s request.

## Footnotes

* Additional information available at http://www.cdc.gov/uscs.

† Population estimates for 2009 incorporate bridged single-race estimates that are derived from the original multiple race categories in the 2000 U.S. Census. Additional information is available at http://seer.cancer.gov/popdata/index.html and http://www.census.gov/popest/topics/methodology.

§ Additional information available at http://seer.cancer.gov/tools/ssm.

¶ Additional information available at http://www.cdc.gov/winnablebattles/tobacco/index.html.

** Additional information available at http://www.fda.gov/tobaccoproducts/guidancecomplianceregulatoryinformation/ucm246129.htm.

†† Additional information available at http://www.cdc.gov/tobacco/stateandcommunity/best_practices/index.htm.

§§ Additional information available at http://www.cdc.gov/cancer/npcr/uscs/technical_notes/interpreting/race.htm.
