# Errata

**Published:** 2014-01-17

## Vol. 63, No. 1

In the report, “Lung Cancer Incidence Trends Among Men and Women — United States, 2005–2009,” multiple errors occurred. On page 2, in the second column, the second full sentence should read, “Lung cancer incidence rates decreased most rapidly among adults aged 35–44 years, decreasing **6.5%** per year among men and **5.8%** per year among women (Table 1).”

On page 3, the title of the Figure should read, “FIGURE. Rate^*^ of invasive lung cancer cases among men and women, by age group **— United States, 2005–2009**.” In the summary box, the last sentence should read, “Continued attention to local, state, and national population-based tobacco prevention and control strategies **is** needed to achieve further reductions in tobacco use among men and women of all ages to reduce lung cancer in the United States.”

On page 3, in Table 1, under the heading Men, the column of APC (%) values, from top to bottom, should read, “−2.6^†^, **0.6,** −**6.5**^†^, −2.9^†^, −4.3^†^, −**2.7**^†^, −1.7.^†^” Under the heading Women, in the column showing RR women to men, the second value should read, “**1.1**”; the column of APC (%) values, from top to bottom, should read, “−1.1^†^, **1.1,** −**5.8**^†^, −0.1, −**3.7**^†^, −0.8, −**0.1**.”

